# Health seeking behaviour among suspected cases of cholera in Cameroonian health districts in Lake Chad basin

**DOI:** 10.1186/s13104-017-2756-9

**Published:** 2017-08-30

**Authors:** Martin Ndinakie Yakum, Jerome Ateudjieu, Etienne Guenou, Ebile Akoh Walter, Malathi Ram, Amanda K. Debes, Anthony Chebe Njimbia, Sonia Sonkeng Nafack, David A. Sack

**Affiliations:** 1 M.A. SANT (Meilleur Accès aux Soins de Santé), P.O. Box 33490, Yaoundé, Cameroon; 2 Department of Biomedical Sciences, University of Dschang, Cameroon, P.O. Box 067, Dschang, Cameroon; 3 Division of Health Operations Research, Ministry of Public Health, Yaoundé, Cameroon; 4 Johns Hopkins University Bloomberg School of Public Health, Baltimore, MD USA

**Keywords:** Health seeking behaviour, Cholera, Diarrhoea, Cameroon

## Abstract

**Background:**

Cholera outbreaks are recurrent in Cameroon and despite the efforts put together during epidemics, they are always associated with a high case fatality. Inadequate demand for health care is one of the major factors that might be responsible for the high case fatality. This study was conducted to describe the health seeking behaviour of suspected cases of cholera in four health districts of the Far North Cameroon.

**Methods:**

We conducted a health facility based descriptive study involving suspected cases of cholera received in health facilities. Data was collected from August 2013 to October 2015 with the help of a questionnaire and analysis done by running frequency and calculating confidence interval at 95% with Epi Info version 3.5.4.

**Results:**

A total of 1849 cases were enrolled, with 997 (53.9%) being males. 534 (28.9%) were children under the age of 5 and 942 (50.9%) were above the age of 14. About 373 (20%) of diarrhoeal patients arrived in the health facility more than 2 days following the onset of diarrhoea, with 916 (50%) of them being seriously dehydrated. Also, about 624 (34%) of these patients had sought treatment elsewhere before coming to the health facility where they were enrolled, and about 86% of them did not received ORS. Taking 2 or more days after diarrhoea onset or taking more than 1 h to travel from home to health facility was associated with severe dehydration in patients.

**Conclusions:**

The delay between the onset of diarrhoea and seeking treatment from a health provider determines the seriousness of suspected cases of cholera in the Far North Cameroon. While conducting an anthropological study to understand reasons why a health provider is not the first option during diarrhoeal episodes, we recommend that a system of community case detection and reference to health facilities should be put in place during cholera outbreaks to minimize its case fatality rate.

**Electronic supplementary material:**

The online version of this article (doi:10.1186/s13104-017-2756-9) contains supplementary material, which is available to authorized users.

## Background

Acute diarrhoea is among the leading causes of death in the world and is more serious in developing countries where access to water, sanitation and hygiene is very limited [1, 2]. It is the number two cause of mortality among children below 5 years and is responsible for 1 in every 5 deaths in children [2–4]. In 2013, the prevalence of diarrhoea in Sub-Saharan Africa was still very high compared to the developed world (about 39.1% against 7.2%) [5, 6]. In Cameroon, the prevalence of diarrhoea in the general population is not known, but the prevalence in children below 5 years was approximately 21.0% in 2011. Acute diarrhoea is caused by a broad spectrum of viral, parasitic and bacterial entero-pathogens, many of which are endemic in Cameroon, and others occurring in the form of outbreaks [5, 7, 8].

Cholera is one of the diarrhoeal diseases that occur in the form of outbreaks in Cameroon and has recently called the attention of the health authorities. This is because of the high recurrences, attack rate during epidemics and the case fatalities [9]. It is one of the priority diseases under epidemiological surveillance at all health facilities, both private and public. Therefore, it is mandatory to report all suspected cases (acute watery diarrhoea with severe dehydration) within 1 week of detection. The reporting is to include a report of zero (0) when no case is detected [10]. The reporting is daily during a cholera epidemic. In addition community sensitisation and other preventive activities, and outreach treatment centres are opened in faraway communities to minimize case fatality rate of the disease. Treatment at these centres includes ORS and medications given by community volunteers and auxiliary nurses. Despite these efforts, the mortality rate remains very high [9].

Also, the treatment options for cholera-like syndrome are very simple [1] and effective, yet the burden of this health event in terms of mortality is still very high in Cameroon [9]. Cameroon has registered numerous cholera outbreaks between 1971 and 2015, 9 of which began in the Far North region [9]. Among the cholera outbreaks to date in Cameroon, the case fatality rate (CFR) has never been lower than 3.8% and sometimes as high as 15% [7]. These case fatality rates are too high compared to what is expected. According to the WHO, if case management is completed correctly, the CFR would be <1% [11]. The objective of the study is to determine if health seeking behaviour of patients suspected of cholera determines the outcome of their illnesses.

Delays in care-seeking and incorrect case management have been identified by many authors to be responsible for high CFR of Diarrhoeal diseases [1, 11–14]. Possible explanations of high CFR in Cameroon can be that patients arrive in the health facilities when their conditions are already critical (late arrival), insufficient number of personnel and insufficient supplies (ORS, ringer, antibiotics etc.) to adequately manage the number of cases arriving at once. The late arrival to health facilities could be because patients first tried an alternative treatment option and only turned to the provider when this failed. Many patients live far from the health facilities and must travel a reasonable distance before reaching the facilities. Poor roads and lack of fast and effective means of transport further inhibits treatment during emergency situations.

This paper aims to describe the health seeking behaviour of diarrhoeal patients that were treated in targeted health facilities. Key factors being assessed include where patients first sought treatment, delay between onset of diarrhoea and the arrival in the hospital and the dehydration state at which patients are received in the hospital. Findings are expected to guide interventions for better cholera control in the specific study area as well as at a national level.

## Methods

### Study design

This was a health facility based cross-sectional study involving suspected cholera cases who sought treatment at health facilities. Data was collected from August, 2013 to October, 2015 as part of the “Sustainable Cholera Surveillance for Cameroon” Project in the Far North region. This project is funded as part of the DOVE (Delivering Oral Cholera Effectively) project by the Bill and Melinda Gates Foundation. All patients suspected of cholera were eligible for the study. Data collection was done with the help of a structured questionnaire administered to patients by study nurses, and data analysis performed with EpiInfo version [3.5.4].

### Study setting

The study was conducted in four Cameroon health districts (Kousseri, Goulfey, Makary and Mada) in the Lake Chad basin. These four health districts have 38 health areas, 40 health facilities (38 public and 2 private) including Kousseri Regional Hospital and a total population of about 600,000 estimated in 2015. This area borders Chad in the east, Nigeria in the West, the Lac Chad in the North and Maga and Bodo health districts in the South. The predominant tribal groups are Arabs and kotoko. This area was selected for the study as it is a hotspot for cholera outbreaks in Cameroon. Seven health facilities were selected based on high case rate during the previous epidemic of cholera in 2011. These facilities treat suspect cases from Cameroon as well as from neighbouring countries including Chad, Niger or Nigeria. All of the study specific health facilities were public.

### Sampling technique

Two surveillance phases (routine and intensive) were implemented in seven targeted health facilities in a 15-day cycle. During routine surveillance (lasting 12/15 days), only patients from 5 years and above, consulting for an acute watery diarrhoea and severe dehydration, were included into the study (WHO case definition of a suspected case of cholera) [13]. In the intensive phase, all patients, irrespective of age, who presented with acute watery diarrhoea and dehydration, were enrolled (lasting 3/15 days) in the study. Patients with bloody stool, chronic diarrhoea (onset more than 7 days before consultation) or with less than three stools within 24 h were excluded from the study.

### Consenting procedures

A written consent form was signed by participants before being included in the study. For minors (all participants less than 21 years), the informed consent was signed by their legal representative.

### Study procedures

The data collection tool was a structured questionnaire developed by the research team and was pretested before usage. A study nurse was recruited from each health facility and trained on the consent and data collection procedures. As previously described, the data collection process was supervised in a cycle of 15 days by field supervisors who were also recruited and trained. The study nurses oversaw patient screening for eligibility and questionnaire administration.

Dehydration status of the patients was evaluated by visual inspection (the general look, eyes, tears, mouth and tongue, and thirst) and by palpation (skin pinch). The determination of dehydration stage of the patient was based on the WHO guidelines for treatment of diarrhoea [15].

### Data analysis

Variables collected in this study include socio-demographic characteristics, initial place sought for treatment (pharmacies, street drugs, traditional healers, community health worker, faith based/NGO/GIC, private health provider, Public Health provider, none), behaviours (self-medication, prescription, no treatment), treatments taken before reaching the hospital (commercial ORS, homemade sugar and salt solution, IV fluids/ringer lactate, none) and progress of the illness. See data collection form (Additional file 1) for details of the data collected. During our data analysis, proportions were calculated by running frequency and calculating confidence intervals at 95%. Data were analysed with EpiInfo software version number 3.5.4.

## Results

### Characteristics of participants

A total of 1849 diarrhoeal patients were enrolled amongst which 997 (53.9%) were males. 944 (51.1%) participants were enrolled during the intensive phase and 905 (48.9) during the routine phase of the study. 534 (28.9%) were children under 5 years and 942 (50.9%) were above the age of 14. 916 (49.5%) patients were severely dehydrated at their arrival in the health facility and the rest were either not dehydrated or moderately dehydrated.

### Health seeking behaviour stratified by age group

Table 1 presents the health seeking behaviour of participants stratified by age groups. It suggests that about 33.8% of diarrhoeal patients first sought treatment elsewhere before arriving at the health facility where they were enrolled in the study. Further, only 8.3% sought previous care from a health provider. Also, 85.8% of the patients presenting to the health facility had not consumed any oral rehydration solution while 31.4% of them had taken antibiotics or other drugs before arriving at the facilities. Moreover, 23% of patients lived far from the health facilities (>5 km) with 20.2% of them presenting to the health facility more than 2 days after the onset of diarrhoea. Even though 1405 (76.0%) patients were driven, up to 22.8% travelled >1 h to reach the facility. Close to 80% of children less than 5 years of age where taken to the study facilities with no prior treatment. The rate of self-medication was lowest in children and progressively increased with increasing age.

**Table 1:** Health seeking behaviour stratified by age group

Indicator	Frequency (%)				P value
	Total	<5 years	5–14 years	>14 years	
Where the patient first sought treatment					0.0000
Pharmacy/other health facilities	132 (7.1)	29 (5.4)	23 (6.2)	80 (8.5)	
Community volunteers/distributors	21 (1.1)	5 (0.9)	4 (1.1)	12 (1.3)	
Auto-medication/unknown medications	451 (24.4)	75 (14.0)	96 (25.7)	280 (29.7)	
Traditional healers	20 (1.1)	3 (0.6)	4 (1.1)	557 (59.1)	
No treatment sought	1225 (66.3)	422 (79.0)	246 (66.0)	557 (59.1)	
Rehydration solution taken at the onset of diarrhoea before coming to the study site					0.0000
Yes	263 (14.2)	34 (6.4)	52 (13.9)	765 (81.2)	
No	1586 (85.8)	500 (93.6)	321 (86.1)	177 (18.8)	
Any medication taken					0.0000
No	1269 (68.6)	422 (79.0)	252 (67.6)	595 (63.2)	
Antibiotic	244 (13.2)	54 (10.1)	33 (8.8)	157 (16.7)	
Others	336 (18.2)	58 (10.9)	88 (23.6)	190 (20.2)	
Duration from symptom onset to the time of seeking treatment (days)					0.0000
≤2	1476 (79.8)	350 (65.5)	299 (80.2)	827 (87.8)	
>2	373 (20.2)	184 (34.5)	74 (19.8)	115 (12.2)	
Distance travelled to reach the health facility (km)					0.0000
<5	1423 (77.0)	355 (66.5)	294 (78.8)	774 (82.2)	
≥5	426 (23.0)	179 (33.5)	79 (21.2)	168 (17.8)	
Time travelled to health facility (h)					0.0000
≤1	1427 (77.2)	445 (83.3)	305 (81.8)	677 (71.9)	
>1	422 (22.8)	89 (16.7)	68 (18.2)	265 (28.1)	
Mode of travel to health facility					0.0000
Motorized	1405 (76.0)	358 (67.0)	287 (76.1)	760 (80.7)	
Non-motorized	444 (24.0)	176 (33.0)	86 (23.1)	182 (19.3)	

### Health seeking behaviour stratified by the dehydration state of the patients

The health seeking behaviour stratified with the dehydration state of the patients is presented in Table 2. From Table 2, patients who had initially sought treatment elsewhere were more severely dehydrated than those who sought care only from the study health facility. Interestingly the rate of severe dehydration was greatly reduced in patients who initially sought treatment from a provider (pharmacy, health facility, community health worker). Also, more severe dehydration was seen in (1) patients who never took an ORS; (2) patients who took antibiotics or other drugs during their first line treatment; (3) patients who arrived to the health facility more than 2 days after diarrhoeal onset; (4) and patients who were not driven to the health facility. However, the result does not identify a significant relationship between the distance travelled to the health facility and the level of dehydration. Rather, it suggests that people who took less than 1 h to reach the facility were more likely to be severely dehydrated that those who took a longer time.

**Table 2:** Health seeking behaviour stratified by severity of diarrhoea (severity based on # of stools and dehydration)

Indicator	Total	Frequency (%)		P value
		Moderate/no dehydration	Severe dehydration	
Where the patient first sought treatment				0.0000
Pharmacy/other health facilities	132 (7.1)	94 (10.1)	38 (4.1)	
Community volunteers/distributors	21 (1.1)	9 (1.0)	12 (1.3)	
Auto-medication/unknown medications	451 (24.4)	38 (4.1)	413 (45.1)	
Traditional healers	20 (1.1)	12 (1.3)	8 (0.9)	
No treatment sought	1225 (66.3)	780 (83.5)	445 (48.6)	
Rehydration solution taken at the onset of diarrhoea before coming to the provider				0.0000
Yes	263 (14.2)	92 (9.9)	171 (18.7)	
No	1586 (85.8)	841 (90.1)	745 (81.3)	
Any medication taken				0.0000
No	1269 (68.6)	728 (78.0)	541 (59.1)	
Antibiotic	244 (13.2)	111 (11.9)	133 (14.5)	
Others	336 (18.2)	94 (10.1)	242 (26.4)	
Duration from symptom onset to the time of seeking treatment (days)				0.0000
≤2	1476 (79.8)	803 (86.1)	673 (73.5)	
>2	373 (20.2)	130 (13.9)	243 (26.5)	
Distance travelled to reach the health facility (km)				0.9084
<5	1423 (77.0)	717 (76.8)	706 (77.1)	
≥5	426 (23.0)	216 (23.2)	210 (22.9)	
Time travelled to health facility (h)				0.0000
≤1	1427 (77.2)	619 (66.3)	808 (88.2)	
>1	422 (22.8)	314 (33.7)	108 (11.8)	
Mode of travel to health facility				0.0000
Motorized	1405 (76.0)	782 (83.8)	623 (68.0)	
Non-motorized	444 (24.0)	151 (16.2)	293 (32.0)	

### Health seeking behaviour stratified by sex

Table 3 shows the health seeking behaviour stratified by sex. From the table, it can be seen that the health seeking behaviour of patients was similar regardless of sex (see Table 3).

**Table 3:** Health seeking behaviour stratified by sex

Indicator	Total	Frequency (%)		P value
		Male	Female	
Where the patient first sought treatment				0.4633
Pharmacy/other health facilities	132 (7.1)	77 (7.7)	55 (6.5)	
Community volunteers/distributors	21 (1.1)	10 (1.0)	11 (1.3)	
Auto-medication/unknown medications	451 (24.4)	251 (25.2)	200 (23.5)	
Traditional healers	20 (1.1)	13 (1.3)	7 (0.8)	
No treatment sought	1225 (66.3)	646 (64.8)	579 (68.0)	
Rehydration solution taken at the onset of diarrhoea before coming to the provider				0.2741
Yes	263 (14.2)	150 (15.0)	113 (13.3)	
No	1586 (85.8)	847 (85.0)	739 (86.7)	
Any medication taken				0.7790
No	1269 (68.6)	679 (68.1)	590 (69.2)	
Antibiotic	244 (13.2)	131 (13.1)	113 (13.3)	
Others	336 (18.2)	187 (18.8)	149 (17.5)	
Duration from symptom onset to the time of seeking treatment (days)				0.9190
≤2	1476 (79.8)	795 (79.7)	681 (79.9)	
>2	373 (20.2)	202 (20.3)	171 (20.1)	
Distance travelled to reach the health facility (km)				0.3580
<5	1423 (77.0)	759 (76.1)	664 (77.9)	
≥5	426 (23.0)	238 (23.9)	188 (22.1)	
Time travelled to health facility (h)				0.5444
≤1	1427 (77.2)	764 (76.6)	663 (77.8)	
>1	422 (22.8)	233 (23.4)	189 (22.2)	
Mode of travel to health facility				0.6161
Motorized	1405 (76.0)	753 (75.5)	652 (76.5)	
Non-motorized	444 (24.0)	244 (24.5)	200 (23.5)	

## Discussion

This paper aims to describe the health seeking behaviour of diarrhoeal patients treated in the targeted health facilities, with particular consideration of variables including where the patients first sought treatment, the delay between onset of diarrhoea and arrival at the health facility and the dehydration status of patients upon arrival to the health facility.

Key findings from the study include: approximately 20% of diarrhoeal patients seek treatment at the health facility more than 2 days after the onset of diarrhoea; and 50% of diarrhoeal patients treated in the facilities were severely dehydrated. Interestingly, approximately 34% of these patients had sought treatment elsewhere prior to coming to the health facility. About 86% of the participants did not take any form of ORS prior to seeking treatment. A relationship was observed between the health facility seeking behaviour of patients and the dehydration state and age of patients.

These results support findings in previously conducted studies where the 30–60% of patients with diarrhoea sought treatments from sources other than medical personnel [16–21]. The health seeking behaviour varies by age of the patients; this has been well described as a product of caregivers who provide more attention to younger children when they are ill than older children [2, 21]. However, these results might underestimate health seeking behaviour among diarrheal patients since only patients who presented to the health facility were enrolled. One consequence of the delay in seeking treatment at a health facility is that patients waste time with unnecessary treatment. This delay may result in serious dehydrate or even death prior to seeking treatment at a health facility [22, 23]. If these alternative providers are all identified in the community, the health system can collaborate with them during cholera epidemic to distribute ORS.

The delay between the onset of diarrhoea and seeking care in this study is sufficient to increase the disease seriousness and the death rate since cholera has the capacity to kill within hours of the onset of symptoms [10, 11]. To the best of our knowledge, previous studies have not considered this element of the disease when describing the health seeking behaviour of patients. However, it is a crucial element to consider because if the first line of treatment is the health personnel, and the patient does not seek care in time, it can still result in death. The delay in seeking care could include, but is not limited to, the following reasons: (1) many patients live far from the health facilities and must travel a reasonable distance before reaching care [2]. For example, 23% of the patients in our study declared that they lived more than 5 km away from the health facilities and 22.8% of the remaining patients stated that they travelled more than 1 h to get to the health facility (2) patients first tried an alternative treatment option and only turned to the health provider when this failed [16–19]. Approximately 34% of the patients sought treatment from a different place prior to coming to the health facility (3) poor roads and lack of fast and effective means of transportation to ensure transportation during emergency situations [2]; and (4) poor knowledge on the part of patients and/or care givers regarding danger signs [19]. Approximately 24% of patients who did not seek treatment elsewhere prior to coming to the health facilities also recorded a delay time prior to seeking treatment in the facilities. As a result, many patients die in the community, while others arrive to the health facility in very critical condition. Many of whom might subsequently die as a result of insufficient personnel or a lack of needed supplies to care for severe cases. For instance, close to 50% of these diarrhoeal patients received were already severely dehydrated, far different from the 20% expected, according to the WHO [2, 23–25]. Understanding reasons why patients do not choose the health facility as their primary source of treatment, educating the caregivers and population about what to do in case of severe diarrhoea might help to reduce the delay time and consequently reduce the death rate from diarrhoea.

One of the shortcomings of this research is that it was health facility based but not also community based. Therefore, we cannot understand why people who are ill in the community did not seek care. Also, data was collected by interviews and the truthfulness of the responses could not be verified, incorporating recall bias. These results provide important insight into the health seeking behaviour of patients who come to the health facilities.

## Conclusions

The rate at which suspected cholera patients seek treatment at health facilities secondary to other providers is high. This, coupled with other factors increases the time between disease onset and life-saving treatment. Consequently, this increases the number of patients presenting to the health facilities severely dehydrated. Importantly, this behaviour varies by age group, where children are most likely to be taken to a provider at the onset of symptoms. In order to reduce the mortality rate from cholera and other severe diarrheal disease, we propose the following recommendations:

- To the district health services
  - identify and collaborate with people treating diarrhoea in the community during cholera epidemics to enhance ORS distribution.
  - reinforce community based surveillance especially during cholera epidemics.
  - sensitize the population and caregivers on the need and use of ORS and/or going to the health provider for diarrhoea treatment.
- To researchers
  - using research to discover reasons why patients prefer to be treated outside of the health facility during diarrhoeal episodes.

## Additional file

**Additional file 1.** Data tool, gives details of data collected for this study.

## Abbreviations

DOVE Project: delivery oral cholera vaccine effectively
M.A.SANTE: Meilleur Accès Aux Soin de Sante
ORS: oral rehydration solution
WHO: World Health Organization
