# Supplementary material for: Health seeking behaviour among suspected cases of cholera in Cameroonian health districts in Lake Chad basin
Source: BMC Res Notes. 2017 Aug 30;10:433. doi: 10.1186/s13104-017-2756-9 (PMC5577771; doi:10.1186/s13104-017-2756-9)
Supplement: Supplementary file 1 — Additional file 1. Data tool, gives details of data collected for this study. [file 13104_2017_2756_MOESM1_ESM.docx]

| **DA_CINICAL DATA FOR PATIENTS WITH DIARRHOEA VERSION 3.0**  ***To be completed by the nurse at time of admission into study*** | | | | | | | |
| --- | --- | --- | --- | --- | --- | --- | --- |
| Patient Initials (Enter first digit of first, middle and last name) | | |  | | | | |
| 1 | Patient study Identification number | |  | | | | Apply printed label here |
| 2 | Date of consultation | | DD / MM / YYYY  / / | | | | |
| 3 | Clinical Facility | | 1= Kousseri, 2=Mada, 3=Ngouma, 4=Maltam, 5=Blangoua, ~~6=Darak~~, 7=Naga,  A=Laquintinie, B=Bonassama, C=New-Bell, D=Nylon | | | |  |
| 4 | Type of Registry | | 1=Reception/Emergency;2=Consultation unit; 3=Hospitalisation; 4=Paediatric unit; 5=Surgery, 6=Maternity, 7=Medical doctor ; 8=Others | | | |  |
| 4a | If Q4=Other, specify | | _______________________________________________________ | | | | |
| 5 | Registry Number | | 1. Registry number 1 2. Registry number 2 | | | | |
| 6 | Patient’s age | | Number of years (If <12 months, enter 000)  ***If Age >1 year, Skip to Q7*** | | | |  |
| 6a | For children <1 years | | If age is less than 1 year, record age in months (00-11) here, otherwise leave blank | | | |  |
| 7 | Sex | | 1=Male, 2=Female | | | |  |
| 8 | Person providing the information | | 1=Self, 2=Mother, 3=Father, 4=Legal Guardian, 5=Sibling, 6=Other  “Self must be at least 10 years old” | | | |  |
| 9 | Village / quarter  (Write name of village/ quarter) | | **____________________________________________________________________________________** | | | | |
| 10 | Where is the village / quarter situated? | | 1=Cameroon, 2=Nigeria, 3=Chad, 4=other  ***If “Other”, answer Q10a, else skip to Q11*** | | | |  |
| 10a | If Q10=Other, specify | | **____________________________________________________________________________________** | | | | |
| 11 | Health Area where the village is situated | | **____________________________________________________________________________________** | | | | |
| 12 | Health District where the Health area is situated | | 1= Kousseri, 2=Mada, 3=Makary, 4=Goulfey,  5=Other,  6=Deido, 7=Bonassama, 8=New-Bell, 9=Nylon | | | |  |
| 12a | If Q12= other, specify  (Write the name of district and country) | | **____________________________________________________________________________________** | | | | |
| **MEDICAL HISTORY** | | | | | | | |
| 13 | Date of onset of diarrhoea | | DD / MM / YYYY  / / | | | | |
| 14 | Onset time for diarrhoea | | 24 hour clock, give approximate | | | | : |
| 15 | General appearance | | 1=Alert, 2=Restless, irritable, 3=Lethargic or unconscious | | | |  |
| 16 | Eyes | | 1=Normal, 2=Sunken, 3=Very sunken | | | |  |
| 17 | Mouth & Tongue | | 1=Moist,2= Dry, 3=Very dry | | | |  |
| 18 | Thirst | | 1=Drinks normally, 2=Drinks eagerly, 3=Drinks poorly or unable to drink | | | |  |
| 19 | Skin elasticity | | 1=pinch goes back quickly, 2= slowly, 3=very slowly | | | |  |
| 20 | Pulse rate | | 1=Normal, 2=Rapid, 3=Feeble, 4=Imperceptible | | | |  |
| 21 | Dehydration status | | 0=None, 1=Some, 2=Moderate/severe | | | |  |
| 22 | Body weight | | 999.9=if not measured  if measured, record weight in kg | | | | . . kg |
| 23 | Fecal specimen obtained? | | 0=Not obtained , 1=Stool, 2=Rectal swab | | | |  |
| 24 | Time fecal specimen obtained | | 24-hour clock | | | | : |
| 25 | Fecal specimen ID | |  | | | | Apply printed label here |
| 26 | Visual appearance of the stool | | 1=Firm, 2=Soft, 3=Very loose, 4=Watery, 5=Like rice-water | | | |  |
| 27 | Any visible blood in the stool? | | 1=Yes, 0=No | | | |  |
| 28 | How many stools in the last 24 hours? | | Record approximate number | | | |  |
| 29 | Vomiting in the last 24 hours? | | Record approximate number | | | |  |
| 30 | Abdominal cramps? | | 0=None, 1=Mild, 2=Moderate, 3=Severe | | | |  |
| 31 | Fever reported? | | 0=None, 1=Feeling feverish, 2=Definite fever, 3=Rigors or chills; 4=Rigors and chills | | | |  |
| 32 | If this patient is below 3 years of age, is he/she breast fed | | 0=No, 1=Partial, 2=Predominant , 3= Exclusive, 4=Not applicable (If age >=3 years) | | | |  |
| 33 | How many days after diarrhoea began did you first seek treatment by a provider? | | Record number | | | |  |
| 34 | Did you first seek treatment at any other health facility? | | 0=No, 1= Traditional healer, 2=Private pharmacy, 3=Community health worker, 4=Other government clinic, 5=Private clinic/provider, 6=Community distributer, 7=Self-, 8=faith-based/NGO/GIC, 9=Unknown, 10=Other | | | |  |
| 34a | If Q34=Other, specify | | **____________________________________________________________________________________** | | | | |
| 35 | Since diarrhoeal episodes began, have you taken or received any rehydration treatment? | | 0=No, 1=Commercial ORS, 2= Homemade salt and sugar solution, 3=IV Fluids/ Ringer’s Lactate | | | |  |
| 36 | Since diarrhoeal episodes began, have you taken any medicines? | | 0=No, 1=Traditional Medicine, 2=Antibiotics, 3=Unknown medicine, 4=Other | | | |  |
| 36a | If Q36=Other, specify | | **____________________________________________________________________________________** | | | | |
| 37 | How many days ago did you start this treatment(s) | | Record number | | | |  |
| 38 | What was the distance travelled to seek treatment at this health facility today? | | 0=<1km, 1=1-4km, 2= 5-9km, 3=10-14km, 4=>14km, 5=Don’t know | | | |  |
| 39 | How long did the travel to the health facility take? | | 1=<1 hour 2=1-4 hours, 3=5-8 hours, 4=>8 hours, 5=Don’t know | | | |  |
| 40 | How did the patient come to the facility for this illness? | | 0=Walked, 1=Bus , 2=Car, 3=Animal, 4=Bicycle, 5=Motorcycle, 6=Ambulance, 7=Other | | | |  |
| 40a | If Q40=Other, specify | | **____________________________________________________________________________________** | | | | |
| 41 | Does the patient know of a case of diarrhea in the past month | | 0=No, 1=Yes, in the family, 2=Yes, in the community, 3=Yes, in the district | | | |  |
| 42 | Did the patient have contact with any known or reported suspect diarrhoea case? | | 0=No, 1=Yes | | | |  |
| 43 | Are any other family members ill with diarrhoea in last 7 days? | | 0=No, 1=Mild illness, 2=Diarrhoea requiring treatment at health facility, 3=Severe diarrhoea requiring IV treatment, 4=Severe diarrhoea leading to death | | | |  |
| **BEHAVIORAL HISTORY** | | | | | | | |
| 44 | Did the patient attend a funeral in the 7 days before becoming ill? | | 0=No, 1=Yes  ***If "No”, skip to Q45*** | | | |  |
| 44a | Date of funeral | | DD / MM / YYYY  / / | | | | |
| 45 | Did the patient attend a social gathering in the 7 days before becoming ill? | | 0=No, 1=Yes  ***If “No”, skip to Q46*** | | | |  |
| 45a | Date of Gathering | | DD / MM / YYYY  / / | | | | |
| 46 | Did the patient attend a market or trading center in the 7 days before becoming ill? | | 0=No, 1=Yes  ***If “No”, skip to Q47*** | | | |  |
| 46a | Date of attendance: | | DD / MM / YYYY  / / | | | | |
| 47 | Did the patient travel outside the home village/town in the last 7 days before becoming ill? | | 0=No, 1=Yes  ***If” No”, skip to Q48*** | | | |  |
| 47a | Date of Travel: | | DD / MM / YYYY  / / | | | | |
| 48 | Did you have soap for hand washing in your house yesterday? | | 0=No, 1=Bar Soap, 2=Liquid/dishwashing soap, 3= Powder/laundry soap/detergent,  4= Other | | | |  |
| 49 | Did you wash your hands with soap at least once in the past 24 hours? | | 0=No, 1=Yes  ***If “No”, skip to Q50*** | | | |  |
| 49a | Did you wash your hands with soap after using the toilet or outside for defecation during last 24 hours? | | 0=No, 1=Yes, 2=Not applicable | | | |  |
| 49b | Did you wash your hands with soap after cleaning children’s bottoms during last 24 hours? | | 0=No, 1=Yes, consistently, 2=Sometimes  3=Not applicable (No children or does not wash children’s bottoms) | | | |  |
| 49c | Did you wash your hands with soap at the time of cooking or food preparation during last 24 hours? | | 0=No, 1=Yes, consistently, 2=Sometimes  3=Not applicable (No children or does not prepare food) | | | |  |
| 49d | Did you wash your hands with soap at the time of feeding children? | | 0=No, 1=Yes, consistently, 2=Sometimes, 3=Not applicable (No children or does not feed children) | | | |  |
| 50 | What type of facilities do you have for hand washing? | | 1=Tap, faucet, 2=Basin or bucket, 3=Bouilloire (Container from which water is poured), 4=Forage pump, 5=Other | | | |  |
| 50a | If Q50=Other, specify | | **____________________________________________________________________________________** | | | | |
| 51 | Main source of drinking water during last week | | 1=Piped/Tap water, 2=Forage pump, 3=Dug well, 4=Pond, 5=River, 6=Lake Chad, 7=Spring, 8=Other | | | |  |
| 51a | If Q51=Other, specify | | **____________________________________________________________________________________** | | | | |
| 52 | Source of water for washing utensils | | 1=Piped/Tap water, 2=Forage pump, 3=Dug well, 4=Pond, 5=River, 6=Lake Chad, 7=Spring, 8=Other | | | |  |
| 52a | If Q52=Other, specify | | **____________________________________________________________________________________** | | | | |
| 53 | Source of water for bathing | | 1=Piped/Tap water, 2=Forage pump, 3=Dug well, 4=Pond, 5=River, 6=Lake Chad, 7=Spring, 8=Other | | | |  |
| 53a | If Q53=Other, specify | | **____________________________________________________________________________________** | | | | |
| 54 | How many minutes is the drinking water source from the house? | | 1= <1 minute, 2= 1-10 minutes; 3= 11-30 minutes, 4=30-60 minutes, 5=> 1 hour ; 6=Don’t know | | | |  |
| 55 | How is water stored at the house | | 1=Closed tank, 2=Closed smaller vessels, 3=Open vessels, 4=No storage of water, 5=Other, 6=Canari(Traditional Storage pot) | | | |  |
| 55a | If Q55=Other, specify | | **____________________________________________________________________________________** | | | | |
| 56 | Is the drinking water used at the house in the treated before drinking? | |  | | | |  |
| 56a | If Q56=Other, specify | | **____________________________________________________________________________________** | | | | |
| 57 | What toilet facility is used by **most** members of your household? | | 0=No facility/bush/field/water body, 1= flush toilet 2=Pit Latrine, 3=Don’t know, 4=Other | | | |  |
| 57a | If Q60=Other, specify | | **____________________________________________________________________________________** | | | | |
| 58 | We do not think that you have cholera, but we would like know about your knowledge about cholera. Can you tell us how to prevent cholera? | | Check all that apply | | a)Washing hand with soap before meals | | |
|  |  | |  | | b)Treating drinking water with chlorine | | |
|  |  | |  | | c)Boiling drinking water | | |
|  |  | |  | | d)Getting vaccinated against cholera | | |
|  |  | |  | | e)Eating heated food | | |
|  |  | |  | | f)Taking medicines | | |
|  |  | |  | | g) Don’t know | | |
|  |  | |  | | h) Other | | |
| 58h1 | If Q58 =Other, specify | | **____________________________________________________________________________________** | | | | |
| 59 | What is the first treatment to take before seeking care in a health facility in case someone suffers from frequent watery diarrhoea? | | 0=None, 1=ORS, 2=Doxicycline, 3=Cotrimoxazol (Bactrim), 4=Methronidazol (Flagyl), 5=Any antibiotics, 6=Traditional medicine, 7=Don’t know, 8=Other | | | |  |
| 59a | If Q62=Other, specify | | **____________________________________________________________________________________** | | | | |
| **SOCIAL HISTORY** | | | | | | | |
| 60 | Tribal group | 1=Arabs; 2= Kotoko; 3= Foulbe; 4 =Haoussa, 5=Kanouri, 6= Others | | | |  | |
| 60a | If Q60=Other, specify | **____________________________________________________________________________________** | | | | | |
| 61 | Religion | 1=Muslim, 2=Christian, 3=Pagan, 4=Other | | | |  | |
| 61a | If Q61=Other, specify | **____________________________________________________________________________________** | | | | | |
| 62 | How many years of schooling have you  had? |  | | | |  | |
| 63 | What is your relationship to the Head of the Household (HH)? | 1=Self, 2=Wife/Husband, 3= Son/Daughter, 4=Son-in-law/Daughter-in-law, 5=Grandchild, 6=Parent, 7=Parent-in-Law, 8=Brother/Sister, 9=Niece/nephew, 10=Other relative, 11=Not related, 12=Don’t know | | | |  | |
| 64 | What is the gender of the HH? | 1=Male, 2=Female | | | |  | |
| 65 | What level of education has HH had? | 1=Never attended school;2=Primary; 3=Secondary;4=University | | | |  | |
| 66 | What occupation does the head of household have? | 0=None, 1=Fisherman, 2= Animal Raising/Cattle Herd, 3=Farmer, 4=Civil Servant, 5=Trader, 6=Housewife, 7=Retired, 8=Other | | | |  | |
| 66a | If Q66=Other, specify | **____________________________________________________________________________________** | | | | | |
| ***If the patient is <10 years old, ask Q67, else skip to Q68*** | | | | | | | |
| 67 | How many years of schooling has the mother or persons caring for patient had? |  | | | |  | |
|  | | | | | | | |
| 68 | What construction material is your house roof made of? | | | 1=Thatch, 2=Metal, 3= Wood, 4=Cement, 5=Other, 6=Mud Clay | |  | |
| 69 | Does your family own a television? | | | 0=No, 1=Yes | |  | |
| 70 | Does your family or someone in your family own a mobile phone? | | | 0=No, 1=Yes | |  | |
| 71 | Does your family or someone in your family own a motorcycle? | | | 0=No, 1=Yes | |  | |
| 72 | Does your house have electricity? | | | 0=No, 1=Yes | |  | |
| 73 | Have you changed the source of your drinking water during the last month? | | | 0=No, 1=Yes | |  | |
| 73a | If the answer is yes, what was your previous water source for drinking? | | | 1=Piped/Tap water, 2=Forage pump, 3=Dug well, 4=Pond, 5=River, 6=Lake Chad, 7=Spring, 8=Other | |  | |
| 73b | If the answer is yes, why did you change your water source? | | | 1=Previous water dried up, 2=Previous water was not clean,3=New water source is closer,4= Liked the new water better, 5=Other,6= Don’t know | |  | |
| 74a | Initials of person completing form | | |  | | | |
| 74b | Date form completed | | | DD / MM / YYYY  / / | | | |
